# Speeding up the classical simulation of Gaussian boson sampling with limited connectivity

**DOI:** 10.1038/s41598-024-58136-1

**Published:** 2024-04-01

**Authors:** Tian-Yu Yang, Xiang-Bin Wang

**Affiliations:** 1 State Key Laboratory of Low Dimensional Quantum Physics, Department of Physics, Tsinghua University, Beijing, 100084 China; 2 Jinan Institute of Quantum Technology, SAICT, Jinan, 250101 China; 3 Shanghai Branch, CAS Center for Excellence and Synergetic Innovation Center in Quantum Information and Quantum Physics, University of Science and Technology of China, Shanghai, 201315 China; 4 International Quantum Academy, Shenzhen, 518048 China; 5 Frontier Science Center for Quantum Information, Beijing, 100084 China

**Keywords:** Optics and photonics, Quantum information, Quantum simulation, Computational science, Information theory and computation, Quantum optics

## Abstract

Gaussian boson sampling (GBS) plays a crucially important role in demonstrating quantum advantage. As a major imperfection, the limited connectivity of the linear optical network weakens the quantum advantage result in recent experiments. In this work, we introduce an enhanced classical algorithm for simulating GBS processes with limited connectivity. It computes the loop Hafnian of an $n \times n$ symmetric matrix with bandwidth *w* in $O(nw2^w)$ time. It is better than the previous fastest algorithm which runs in $O(nw^2 2^w)$ time. This classical algorithm is helpful on clarifying how limited connectivity affects the computational complexity of GBS and tightening the boundary for achieving quantum advantage in the GBS problem.

## Introduction

Gaussian boson sampling (GBS) is a variant of boson sampling (BS) that was originally proposed to demonstrate the quantum advantage^1–4^. In recent years, great progress has been made in experiments on GBS^5–11^. Both the total number of optical modes and detected photons in GBS experiments have surpassed several hundred^7,8^. Moreover, it is experimentally verified that GBS devices can enhance the classical stochastic algorithms in searching some graph features^10,11^.

The central issue in GBS experiments is to verify the quantum advantage of the result. The time cost of the best known classical algorithm for simulating an ideal GBS process grows exponentially with the system size^12^. Therefore a quantum advantage result might be achieved when the system size is large enough^13–15^.

However, there are always imperfections in real quantum setups, and hence the time cost of corresponding classical simulation will be reduced. When the quantum imperfection is too large, the corresponding classical simulation methods can work efficiently^16–18^. In this situation, a quantum advantage result won’t exist even if the system size of a GBS experiments is very large. Therefore, finding better methods to classically simulate the imperfect GBS process is rather useful in exploring the tight criteria for quantum advantage of a GBS experiment.

Recent GBS experiments have been implemented using optical circuits with relatively shallow depths^5,7,17,18^. A shallow optical circuit might have limited connectivity and its transform matrix will deviate from the global Haar-random unitary^18,19^. However, the original GBS protocol^2,3^ claims that the unitary transform matrix *U* of the passive linear optical network should be randomly chosen from Haar measure. Classical algorithms can take advantage of the limited connectivity and the deviation from the global Haar-random unitary to realize a speed-up in simulating the whole sampling process^17,18^. Actually, with limited connectivity in the quantum device, the speed-up of corresponding classical simulation can be exponential^18^.

The most time-consuming part of simulating the GBS process with limited connectivity is to calculate the loop Hafnian of banded matrices. A classical algorithm to calculate the loop Hafnian of a banded $n\times n$ symmetric matrix with bandwidth *w* in time $O\left( nw 4^w\right)$ is given in Ref.^17^. Later, an algorithm that takes time $O\left( nw^2 2^w\right)$ is given in Ref.^18^. Here we present a classical algorithm to calculate the loop Hafnian of a banded $n\times n$ matrix with bandwidth *w* in time $O\left( nw 2^w\right)$. We also show that this algorithm can be used to calculate the loop Hafnian of sparse matrices.

Our algorithm reduces the time needed for classically simulating the GBS process with limited connectivity. This is helpful in clarifying how limited connectivity affects the computational complexity of GBS and tightening the boundary of quantum advantage in the GBS problem.

This article is organized as follows. In “Overview of Gaussian boson sampling and limited connectivity” section, we give an overview of the background knowledge which will be used later. In “Simulate the sampling process with limited connectivity” section, we give our improved classical algorithm for calculating the loop Hafnian of banded matrices. Finally, we make a summary in “Summary” section.

## Overview of Gaussian boson sampling and limited connectivity

### Gaussian boson sampling protocol

**Figure 1:**
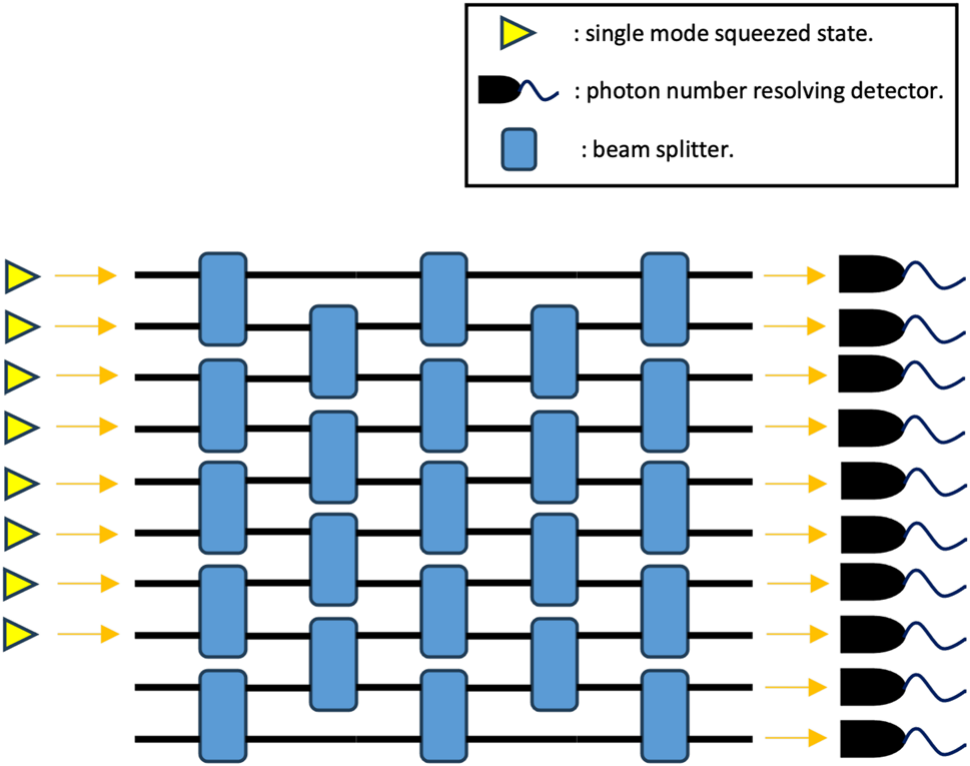
A schematic setup of Gaussian boson sampling. In this example, $K=8$ single-mode squeezed states are injected into a passive linear optical network with $M=10$ optical modes. Then photon number resolving detectors detect the photon number in each output mode. An output pattern ${\bar{n}}=n_1 n_2\ldots n_M$ is generated according to the detected results.

In the GBS protocol, *K* single-mode squeezed states (SMSSs) are injected into an *M*-mode passive linear optical network and detected in each output mode by a photon number resolving detector. The detected photon number of each photon number resolving detector forms an output sample which can be denoted as ${\bar{n}}=n_1 n_2 \ldots n_M$. The schematic setup of Gaussian boson sampling is shown in Fig. 1.

Before detection, the output quantum state of the passive linear optical network is a Gaussian quantum state^20–23^. A Gaussian state is fully determined by its covariance matrix and displacement vector. Denote operator vector ${\hat{\xi }} = ({\hat{a}}_1^\dagger ,\dots ,{\hat{a}}_M^\dagger ,{\hat{a}}_1,\dots ,{\hat{a}}_M)^{T}$, where ${\hat{a}}_i^\dagger$ and ${\hat{a}}_i$ are the creation and annihilation operators in the *i*th ($i\in \left\{ 1,2,\dots ,M\right\}$) optical mode, respectively. The covariance matrix $\sigma$ and displacement vector ${\bar{r}}$ of the Gaussian state ${\hat{\rho }}$ is defined as

1 $$\begin{aligned} \begin{aligned}{}&{\bar{r}} = {\text{Tr}}[{\hat{\rho }} {\hat{\xi }}],\\&\sigma =\frac{1}{2}{\text{Tr}}\left[ {\hat{\rho }}\left\{ ({\hat{\xi }}-{\bar{r}}),({\hat{\xi }}-{\bar{r}})^{\dagger }\right\} \right] ,\\ \end{aligned} \end{aligned}$$

where

2 $$\begin{aligned} \begin{aligned} \left\{ ({\hat{\xi }}-{\bar{r}}),({\hat{\xi }}-{\bar{r}})^{\dagger }\right\}&= ({\hat{\xi }}-{\bar{r}}) ({\hat{\xi }}-{\bar{r}})^{\dagger } \\&\quad + (({\hat{\xi }}-{\bar{r}}) ({\hat{\xi }}-{\bar{r}})^{\dagger })^{\dagger }. \end{aligned} \end{aligned}$$

Notice that $({\hat{\xi }}-{\bar{r}}) ({\hat{\xi }}-{\bar{r}})^{\dagger }$ is the outer product of column vector $({\hat{\xi }}-{\bar{r}})$ and row vector $({\hat{\xi }}-{\bar{r}})^{\dagger }$ and $({\hat{\xi }}-{\bar{r}}) ({\hat{\xi }}-{\bar{r}})^{\dagger } \ne (({\hat{\xi }}-{\bar{r}}) ({\hat{\xi }}-{\bar{r}})^{\dagger })^{\dagger }$ as the operators in the vector ${\hat{\xi }}$ might not commute with each other. As an example, assume $M=1$ so that ${\hat{\xi }}=({\hat{a}}_1^\dagger ,{\hat{a}}_1)^T$, we have

3 $$\begin{aligned} ({\hat{\xi }}-{\bar{r}})({\hat{\xi }}-{\bar{r}})^{\dagger }=\left( \begin{array}{cc} {\hat{a}}_1^\dagger {\hat{a}}_1 &{}\quad {\hat{a}}_1^\dagger {\hat{a}}_1^\dagger \\ {\hat{a}}_1 {\hat{a}}_1 &{}\quad {\hat{a}}_1 {\hat{a}}_1^\dagger \end{array}\right) . \end{aligned}$$

But,

4 $$\begin{aligned} (({\hat{\xi }}-{\bar{r}})({\hat{\xi }}-{\bar{r}})^{\dagger })^{\dagger }=\left( \begin{array}{cc} {\hat{a}}_1 {\hat{a}}_1^\dagger &{}\quad {\hat{a}}_1^\dagger {\hat{a}}_1^\dagger \\ {\hat{a}}_1 {\hat{a}}_1 &{}\quad {\hat{a}}_1^\dagger {\hat{a}}_1 \end{array}\right) \ne ({\hat{\xi }}-{\bar{r}})({\hat{\xi }}-{\bar{r}})^{\dagger }. \end{aligned}$$

Usually, a matrix (say matrix *A*) is used to calculate the output probability distribution of a GBS process^2,3,14^. Denote that $$X_{2M} = \left( \begin{array}{cc} 0 &{}\quad I_M \\ I_M &{}\quad 0 \end{array}\right)$$, and $I_{2M}$ (or $I_M$) as identity matrix with rank 2*M* (or *M*). The matrix *A* is fully determined by the output Gaussian sate as follows:

5 $$\begin{aligned} A_{i,j}={\left\{ \begin{array}{ll}({\tilde{A}})_{i, j} &{}\quad {\text{if }} i \ne j \\ {\tilde{y}}_i &{}\quad {\text{if }} i=j\end{array}\right. }, \end{aligned}$$

where

6 $$\begin{aligned} \begin{aligned}{}&\sigma _Q=\sigma +I_{2 M} / 2, \\ {}&{\tilde{A}}=X_{2 M}\left( I_{2 M}-\sigma _Q^{-1}\right) , \\ {}&{\tilde{y}}= X_{2 M}\sigma _Q^{-1} {\bar{r}}. \end{aligned} \end{aligned}$$

The probability of generating an output sample ${\bar{n}}$ is

7 $$\begin{aligned} \begin{aligned}{}&p({\bar{n}})=\frac{\exp \left( -\frac{1}{2} {\bar{r}}^{\dagger } \sigma _Q^{-1} {\bar{r}} \right) }{\sqrt{{\text{det}}(\sigma _Q)}} \frac{{\text{lhaf}}\left( A_{{\bar{n}}}\right) }{n_{1} ! \cdots n_{M} !},\\ \end{aligned} \end{aligned}$$

where ${\text{lhaf}}(A)=\sum _{M \in {\text{SPM}}(n)} \prod _{(i, j) \in M} A_{i, j}$ is the loop Hafnian function of matrix *A*, and ${\text{SPM}}(n)$ is the set of single-pair matchings^12^ which is the set of perfect matchings of complete graph with loops on *n* vertices. The matrix $A_{{\bar{n}}}$ is obtained from *A* as follows ${\bar{n}}$^14,24^: for $\forall i = 1,\dots , M$, if $n_i = 0$, the rows and columns *i* and $i+M$ are deleted from the matrix *A*; if $n_i \ne 0$, rows and columns *i* and $i+M$ are repeated $n_i$ times.

### Continuous-variables quantum systems

If a Gaussian state is input into a linear optical network, the output quantum state is also a Gaussian state. Denote the unitary operator corresponding to the passive linear optical network as ${\hat{U}}$. A property of the passive linear optical network is:

8 $$\begin{aligned} {\hat{U}}^\dagger {\hat{\xi }} {\hat{U}} = T {\hat{\xi }}, \end{aligned}$$

where

9 $$\begin{aligned} T = \left( \begin{array}{cc} U &{}\quad 0 \\ 0 &{}\quad U^* \end{array}\right) . \end{aligned}$$

The output Gaussian state and the input Gaussian state is related by

10 $$\begin{aligned} \begin{aligned} \sigma&=T \sigma _{\text{in}} T^{\dagger },\\ {\bar{r}}&= T{\bar{r}}_{\text{in}},\\ \end{aligned} \end{aligned}$$

where $\sigma _\text{in}$ and ${\bar{r}}_{\text{in}}$ are the covariance matrix and displacement vector of the input Gaussian state. The SMSS in input mode *i* has squeezing strength $s_i$ and phase $\phi _i$. For simplicity and without loss of generality, assume that $\phi _i = 0$ for $i = 1,\dots ,M$. The covariance matrix of the input Gaussian state is

11 $$\begin{aligned} \sigma _{in} = \frac{1}{2}\left( \begin{array}{cc} \bigoplus \limits _{i=1}^M \cosh 2s_i &{}\quad \bigoplus \limits _{i=1}^M \sinh 2s_i\\ \bigoplus \limits _{i=1}^M \sinh 2s_i &{}\quad \bigoplus \limits _{i=1}^M \cosh 2s_i \end{array} \right) . \end{aligned}$$

The matrix ${\tilde{A}}$ is thus ${\tilde{A}} = {\tilde{B}} \bigoplus \tilde{B^*}$, where

12 $$\begin{aligned} {\tilde{B}} = U \left( \bigoplus \limits _{i=1}^M \tanh s_i \right) U^T. \end{aligned}$$

If a part of the optical modes in the Gaussian state are measured with photon number resolving detectors and the outcome is not all-zero, the remaining quantum state will be a non-Gaussian state^25,26^. An *M*-mode coherent state^20–22,27^ is denoted as $| \vec {\alpha } \rangle$ (or $|\vec {\beta }\rangle$), where $\vec {\alpha } = \left( \alpha _{1},\dots ,\alpha _{M}\right) ^{\mathrm{T}}$ (or $\vec {\beta } = \left( \beta _{1},\ldots ,\beta _{M}\right) ^{\mathrm{T}}$) and $\alpha _i$ (or $\beta _{i}$) for $i=1,\dots ,M$ are complex variables.

A Gaussian state can be represented in the following form:

13 $$\begin{aligned} \begin{aligned} {\hat{\rho }}&= \frac{1}{\pi ^{2M}} \int \mathrm{d}^2 \vec {\alpha } \int \mathrm{d}^2 \vec {\beta } |\vec {\beta }\rangle \langle \vec {\alpha }| \langle \vec {\beta } | {\hat{\rho }} | \vec {\alpha } \rangle \\ {}&= \frac{{\mathscr {P}}_0}{\pi ^{2M}} \int \mathrm{d}^2 \vec {\alpha } \int \mathrm{d}^2 \vec {\beta } |\vec {\beta }\rangle \langle \vec {\alpha }| {\exp } \left( -\frac{|{\tilde{\lambda }}|^2}{2} + \frac{1}{2} {\tilde{\lambda }}^{\mathrm{T}} {\tilde{A}} {\tilde{\lambda }} +{\tilde{\lambda }}^{\mathrm{T}}{\tilde{y}} \right) , \end{aligned} \end{aligned}$$

where

14 $$\begin{aligned} \begin{aligned} {\tilde{\lambda }}&= \left( \beta _{1}^*,\ldots ,\beta _{M}^*, \alpha _{1},\dots ,\alpha _{M}\right) ^{\mathrm{T}},\\ {\mathscr {P}}_0&=\frac{\exp \left( -\frac{1}{2} {\bar{r}}^{\dagger } \sigma _Q^{-1} {\bar{r}} \right) }{\sqrt{{\text{det}}(\sigma _Q)}}. \end{aligned} \end{aligned}$$

For our convenience, define the permutation matrix *P*, such that ${\tilde{\gamma }} = P {\tilde{\lambda }} = \left( \beta _{1}^*,\alpha _{1},\beta _{2}^*,\alpha _{2},\dots ,\beta _{M}^*, \alpha _{M}\right) ^{\mathrm{T}}$. Let ${\tilde{R}} = P^\mathrm{T}{\tilde{A}}P$, ${\tilde{l}} = P {\tilde{y}}$. We then have

15 $$\begin{aligned} {\hat{\rho }} = \frac{{\mathscr {P}}_0}{\pi ^{2M}} \int \mathrm{d}^2 \vec {\alpha } \int \mathrm{d}^2 \vec {\beta } |\vec {\beta }\rangle \langle \vec {\alpha }| {\exp } \left( -\frac{|{\tilde{\gamma }}|^2}{2} + \frac{1}{2} {\tilde{\gamma }}^{\mathrm{T}} {\tilde{R}} {\tilde{\gamma }} +{\tilde{\gamma }}^{\mathrm{T}}{\tilde{l}} \right) . \end{aligned}$$

Suppose the first *N* modes of an *M*-mode Gaussian state are measured and a sample pattern ${\bar{n}}=n_1 n_2 \dots n_N$ is observed. Denote that

16 $$\begin{aligned} \begin{aligned} {\tilde{\gamma }}_h&= \left( {\tilde{\gamma }}_{2N+1}^*,{\tilde{\gamma }}_{2N},\dots ,{\tilde{\gamma }}_{2M}\right) ^{\mathrm{T}},\\ {\tilde{\gamma }}_d&= \left( {\tilde{\gamma }}_1, {\tilde{\gamma }}_2, \dots ,{\tilde{\gamma }}_{2N}\right) ^{\mathrm{T}}, \\ {\tilde{l}}_h&= \left( {\tilde{l}}_{2N+1},{\tilde{l}}_{2N}\dots ,{\tilde{y}}_{2M}\right) ^T,\\ {\tilde{l}}_d&= \left( {\tilde{l}}_{1},{\tilde{l}}_{2}\dots ,{\tilde{l}}_{2N}\right) ^T,\\ {\tilde{R}}&=\left( \begin{array}{cc} {\tilde{R}}_{dd} &{}\quad {\tilde{R}}_{dh}\\ {\tilde{R}}_{hd} &{}\quad {\tilde{R}}_{hh} \end{array}\right) , \end{aligned} \end{aligned}$$

where ${\tilde{R}}_{dd}$ is a $2N\times 2N$ matrix corresponding to modes 1 to *N*, ${\tilde{R}}_{hh}$ is a $2(M-N) \times 2(M-N)$ matrix corresponding to modes $N+1$ to *M*, ${\tilde{R}}_{dh}$ is a $2N \times 2(M-N)$ matrix represents the correlation between modes 1 to *N* and modes $N+1$ to *M*. The remaining non-Gaussian state is^26^

17 $$\begin{aligned} \begin{aligned} {\hat{\rho }}^{({\bar{n}})}&=\frac{{\mathscr {P}}_0}{\pi ^{2 (M-N)}} \int d^2 \vec {\alpha }_{(N)} \int d^2 \vec {\beta }_{(N)} |\vec {\beta }_{(N)} \rangle \langle \vec {\alpha }_{(N)} | F\left( {\tilde{\gamma }}_h\right) , \end{aligned} \end{aligned}$$

where

18 $$\begin{aligned} \begin{aligned} d^2 \vec {\beta }_{(N)}&= d^2 \beta _{N+1} d^2 \beta _{N+2}\dots d^2\beta _{M},\\ d^2 \vec {\alpha }_{(N)}&= d^2\alpha _{N+1} d^2 \alpha _{N+2}\dots d^2 \alpha _{M}, \end{aligned} \end{aligned}$$

$|\vec {\beta }_{(N)}\rangle = |\beta _{N+1},\beta _{N+2}\dots ,\beta _{M}\rangle$ and $|\vec {\alpha }_{(N)}\rangle = |\alpha _{N+1},\alpha _{N+2}\dots ,\alpha _{M}\rangle$ are coherent states, and

19 $$\begin{aligned} \begin{aligned} F\left( {\tilde{\gamma }}_h\right)&=\frac{{\mathscr {P}}_0}{{\bar{n}} !} \exp \left( L_2\right) \prod _{k=N+1}^M\left( \frac{\partial ^2}{\partial \alpha _k \partial \beta _k^*}\right) ^{n_k} \exp \left( L_3\right) \Bigg |_{{\tilde{\gamma }}_d=0}, \\ L_2&=-\frac{1}{2}\left| {\tilde{\gamma }}_h\right| ^2+\frac{1}{2} {\tilde{\gamma }}_h^{T} {\tilde{R}}_{h h} {\tilde{\gamma }}_h+{\tilde{\gamma }}_h^{T} {\tilde{l}}_h, \\ L_3&= -\left| {\tilde{\gamma }}_d\right| ^2+\frac{1}{2} {\tilde{\gamma }}_d^{T} {\tilde{R}}_{d d} {\tilde{\gamma }}_d+{\tilde{\gamma }}_d^{T} {\tilde{l}}_d+{\tilde{\gamma }}_d^{T} {\tilde{R}}_{d h} {\tilde{\gamma }}_h . \end{aligned} \end{aligned}$$

If all optical modes of the Gaussian state are measured (N = M), then Eq. (17) gives the probability of obtaining the output sample ${\bar{n}}$. In this case, the result of Eq. (17) coincides with that of Eq. (7).

### Limited connectivity

As specified in the Gaussian boson sampling protocol^2,3^, the transform matrix *U* of the passive linear optical network should be randomly chosen from the Haar measure. The circuit depth needed to realize an arbitrary unitary transform in a passive linear optical network is *O*(*M*), where *M* is the number of optical modes^19,28–30^. However, due to the photon loss, the circuit depth of the optical network might not be deep enough to meet the requirements of the full connectivity and the global Haar-random unitary^19^. This is because photon loss rate $\varepsilon$ will increase exponentially with the depth of the optical network, i.e., $\varepsilon = \varepsilon _0^D$, where $\varepsilon _0$ is the photon loss of each layer of the optical network. If the photon loss rate is too high, the quantum advantage result of GBS experiments will be destroyed^16,31,32^.

**Figure 2:**
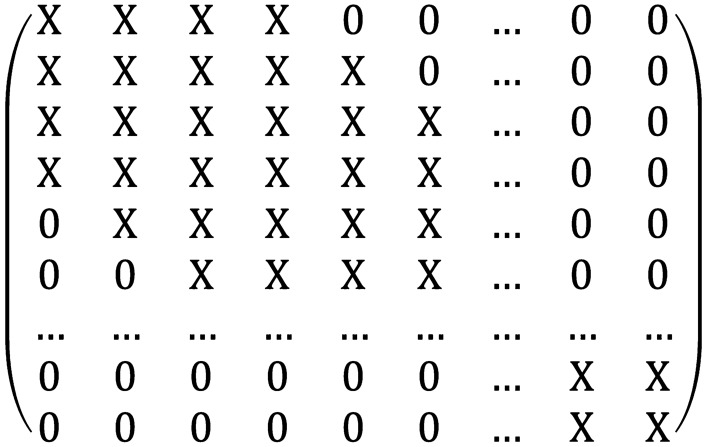
A symmetric matrix with a banded structure. X represents an arbitrary non-zero entry in the matrix. The bandwidth in this example is $w = 3$.

The shallow circuit depth leads to a limited connected interferometer^17–19^. As an example, consider the case where the beam splitters in the optical network is local, which means they act on neighbouring modes, assuming a 2D structure. This is shown in Fig. 1. If $D<M$, the transform matrix *U* of the passive linear optical network will have a banded structure^17^, i.e.,

20 $$\begin{aligned} \left| U_{i,j}\right| =0 \quad \text{for} \ \left| i-j \right| >w_U, \end{aligned}$$

and

21 $$\begin{aligned} \left| U_{i,j}\right| \ne 0 \quad \text{for} \ \left| i-j \right| \le w_U, \end{aligned}$$

where $w_U= D$ is the bandwidth of the transform matrix *U*. An example of a matrix with banded structure is shown in Fig. 2. According to Eqs (10) and (11), the covariance matrix $\sigma$ of the output Gaussian state will have a banded structure. Thus, if beam splitters are local, the circuit depth *D* must be no less than *M*/2 to reach the full connectivity. Note that, according to Eq. (12), matrix ${\tilde{B}}$ [and *A* in Eq. (7)] has bandwidth

22 $$\begin{aligned} w = \left\{ \begin{array}{ll} 2 w_D -1 &{}\quad \text{for} \ w_D < M/2 \\ M - 1 &{}\quad \text{for} \ w_D \ge M/2 \end{array}\right. . \end{aligned}$$

Recently, a scheme known as “high-dimensional GBS” has been proposed^4^. This scheme suggests that by interfering non-adjacent optical modes, the connectivity can be improved while maintaining a relatively shallow circuit depth. However, due to the limited circuit depth, the transformation matrix in this scheme cannot represent an arbitrary unitary matrix. Consequently, the transformation matrix deviates from the global Haar-random unitary. To address this, the scheme introduces a local Haar-random unitary assumption, which says that the transformation matrix corresponding to each individual beam splitter is randomly selected from the Haar measure. Under this local Haar-random unitary assumption, Ref.^18^ demonstrates that when the circuit depth is too shallow, the high dimensional GBS process can be approximate by a limited connected GBS process with a small error.

As a result of the limited connectivity and the deviation from the Haar measure, a speed-up can be realized in simulating the corresponding GBS process^17,18^. The speed-up is attributed to the faster computation of the loop Hafnian for matrices with bandwidth as compared to the computation of general matrices.

### Classical simulation of GBS

To date, the most efficient classical simulation method for simulating a general GBS process has been presented in Ref.^14^. This classical algorithm, which samples from an *M*-mode Gaussian state ${\hat{\rho }}$, operates as follows.

1. If ${\hat{\rho }}$ is a mixed state, it can be decomposed as a classical mixture of pure Gaussian states. We randomly select a pure displaced Gaussian state based on this classical mixture. This can be done in polynomial time^14,21^. Its covariance matrix and displacement vector are denoted as $\sigma$ and ${\bar{r}}$, respectively.
2. If ${\hat{\rho }}$ is a pure state, denote its covariance matrix and displacement vector as $\sigma$ and ${\bar{r}}$, respectively.

For $$k=1,\dots ,M$$:

3. If $k=1,$ drawn a sample ${\bar{\alpha }}^1 = \left( a_{2+1},\dots ,\alpha _{M}\right)$ from the probability distribution:
  23 $$\begin{aligned} p({\bar{\alpha }}) = \frac{1}{\pi ^{M-1}}\left\langle {\bar{\alpha }}^1 \left| {\hat{\rho }}_{d}\right| {\bar{\alpha }}^1 \right\rangle , \end{aligned}$$
  where ${\hat{\rho }}_{d} = {\text{Tr}}_{1} \left[ {\hat{\rho }}\right]$, and ${\text{Tr}}_{1}$ $\left[ {\cdot }\right]$ is the partial trace of mode 1. This process is equivalent to measure the modes $2$ to *M* by heterodyne measurements.
4. Let $\bar{\alpha}^k = \left(\alpha_{k+1},\dots,\alpha_{M}\right)$. Provided that the heterodyne measurements in modes $k+1\;\text{to}\;M$gives $\bar{\alpha}^k$, compute the conditional covariance matrix $\sigma _{1-k}^{({\bar{\alpha }}^k)}$ and displacement ${\bar{r}}_{1-k}^{({\bar{\alpha }}^k)}$ of the remaining Gaussian state. If $k=M$, let $\sigma _{1-k}^{({\bar{\alpha }}^k)} = \sigma$ and ${\bar{r}}_{1-k}^{({\bar{\alpha }}^k)}={\bar{r}}$. Notice that the conditional quantum state in modes $1,\dots ,k$ is still Gaussian if modes $k+1,\dots ,M$ is measured by heterodyne measurements^21^.
5. Given a cutoff $N_{\text{max}}$, use Eq. (7) with $\sigma _{1-k}^{({\bar{\alpha }}^k)}$ and ${\bar{r}}_{1-k}^{({\bar{\alpha }}^k)}$ to calculate $p\left( n_1,\dots ,n_k\right)$ for $n_k = 0,1,\dots ,N_{\text{max}}$.
6. Sample $$n_k$$ from:
  24 $$\begin{aligned} p(n_k) = \frac{p\left( n_1,\dots ,n_k\right) }{p\left( n_1,\dots ,n_{k-1}\right) }. \end{aligned}$$

The most time-consuming part for the above classical simulation method is to calculate the probability $p\left( n_1,\dots ,n_k\right)$ of an output sample pattern $n_1,\dots ,n_M$. According to Eq. (7), we have

25 $$\begin{aligned} \begin{aligned} p\left( n_1,\dots ,n_k\right)&= \frac{\exp \left( -\frac{1}{2} {\bar{r}}_{{\mathscr {A}}}^{({\mathscr {B}})\dagger } (\sigma _Q)_{{\mathscr {A}}}^{({\mathscr {B}})-1} {\bar{r}}_{{\mathscr {A}}}^{({\mathscr {B}})} \right) }{\sqrt{{\text{det}}\left( (\sigma _Q)_{{\mathscr {A}}}^{({\mathscr {B}})}\right) }} \\ {}&\quad \times \frac{{\text{lHaf}}\left( \left( A_{{\mathscr {A}}}^{({\mathscr {B}})}\right) _{{\bar{n}}}\right) }{n_{1} ! \cdots n_{M} !},\\ \end{aligned} \end{aligned}$$

where the ${\bar{r}}_{{\mathscr {A}}}^{({\mathscr {B}})}$, $(\sigma _Q)_{{\mathscr {A}}}^{({\mathscr {B}})}$ and $A_{{\mathscr {A}}}^{({\mathscr {B}})}$ are the corresponding conditional matrices for subsystem contains modes 1 to *k* (denoted as ${\mathscr {A}}$) when modes $k+1$ to *M* are measured by heterodyne measurements with outcome denoted as ${\mathscr {B}}$. $A_{{\mathscr {A}}}^{({\mathscr {B}})}$ can be computed by the following equation:

26 $$\begin{aligned} A_{{\mathscr {A}}}^{({\mathscr {B}})} = {\left\{ \begin{array}{ll}\left( {\tilde{A}}_{{\mathscr {A}}}^{({\mathscr {B}})}\right) _{i, j} &{}\quad {\text{ if } } i \ne j \\ \left( {\tilde{y}}_{{\mathscr {A}}}^{({\mathscr {B}})}\right) _i &{}\quad {\text{ if } } i=j\end{array}\right. }. \end{aligned}$$

Note that if the Gaussian state ${\hat{\rho }}$ is a pure state, the conditional Gaussian state is still a pure state. So, we have ${\tilde{A}}_{{\mathscr {A}}}^{({\mathscr {B}})} = {\tilde{B}}_{{\mathscr {A}}} \bigoplus {\tilde{B}}_{{\mathscr {A}}}^*$. As pointed in Ref.^18^, we have ${\tilde{B}}_{{\mathscr {A}}} = [U (\bigoplus \nolimits _{i} \tanh s_i ) U^T ]_{{\mathscr {A}}}$. This shows that ${\tilde{B}}_{{\mathscr {A}}}$ has the same banded structure as ${\tilde{B}}$. A classical algorithm to calculate the loop Hafnian of a banded matrix is thus needed for the classical simulation of GBS with limited connectivity.

## Simulate the sampling process with limited connectivity

### Loop Hafnian algorithm for banded matrices

An algorithm to calculate the loop Hafnian of an $n \times n$ symmetric matrix with bandwidth *w* in time $O\left( nw 4^w\right)$ is given in Ref.^17^. Then, a faster algorithm with time complexity $O\left( n w_t^2 2^{w_t}\right)$ to calculate the loop Hafnian of an $n\times n$ symmetric matrix is proposed in Ref.^18^, where $w_t$ is the treewidth of the graph corresponding to the matrix^33^. For a banded matrix, the smallest treewidth $w_t$ is equal to the bandwidth *w*, i.e., $w = w_t$. So the time complexity for this algorithm to calculate the loop Hafnian of an $n\times n$ symmetric matrix with bandwidth *w* is $O\left( n w^2 2^w\right)$. Here we show that the loop Hafnian of an $n \times n$ symmetric matrix with bandwidth *w* can be calculated in $O\left( n w 2^w \right)$.

Our algorithm to calculate the loop Hafnian for banded matrices is outlined as follows.

**Algorithm.** To calculate the loop Hafnian of an $n\times n$ symmetric matrix *B* with bandwidth *w*:

1. Let $$C_{\emptyset }^0 = 1$$.

For $$t=1,\dots ,n$$:

2. Let $t_w = \min \left( t+w,n\right)$, and $P(\{t+1,\dots ,t_w\})$ be the set of all subsets of $\left\{ t+1,\dots ,t_w\right\}$.
3. For every $Z^t \in P(\{t+1,\dots ,t_w\})$ satisfying $Z^t \ne \emptyset$ and $\left| Z^t\right| \le \min \left( t,w\right)$, let
  27 $$\begin{aligned} \quad \quad C^t_{Z^t} = \sum _{x \in Z^t } B_{t,x} C^{t-1}_{Z^t \backslash \{x\}} + C^{t-1}_{Z^t \cup \left\{ t\right\} } + B_{t,t} C^{t-1}_{Z^t}, \end{aligned}$$
  and if $t_w \in Z^t$, then
  28 $$\begin{aligned} C^t_{Z^t} = B_{t,t_w} C^{t-1}_{Z^t \backslash \{t_w\}}. \end{aligned}$$
  During the above iterations, if $C^{t-1}_{\{\dots \}}$ is not given in the previous steps, it is treated as 0.
4. Let
  29 $$\begin{aligned} C^t_\emptyset = B_{t,t}C^{t-1}_\emptyset + C^{t-1}_{\{t\}}. \end{aligned}$$

The loop Hafnian of matrix *B* is obtained in the final step $t = N$ by

30 $$\begin{aligned} {\text{lhaf}}\left( B \right) = C^n_{\emptyset }. \end{aligned}$$

An example of calculating the loop Hafnian of a $4 \times 4$ matrix with bandwidth $w = 1$ using our algorithm can be found in Appendix A. Note that, our algorithm for calculating loop Hafnian function of matrices with banded structure can be easily extended to cases where the matrices is sparse (but not banded). A description of this can be found in Appendix B. The time complexity of the above algorithm is $O(nw 2^{w})$ as shown in Theorem 1.

#### Theorem 1

Let *B* be an $n\times n$ symmetric matrix with bandwidth *w*. Then its loop Hafnian can be calculated in $O(nw 2^{w})$.

#### Proof

As shown in our algorithm, the number of coefficients ($C^t_{Z_t}$, $C^t_\emptyset$ and $C^t_{\{t\}}$) needed to be calculated for each $t \in \{1,\dots ,n\}$ is at most $2^{w}$. As shown in Eqs. (27)–(30), in each iteration, we need *O*(*w*) steps to calculate each coefficient $C^{t}_{Z^t}$. So, for each *t*, the algorithm takes $O(w 2^{w})$ steps. The overall cost is thus $O\left( n w 2^{w} \right)$. $\square$

Combining our algorithm with the classical sampling techniques described in Ref.^14^, as summarized in “Classical simulation of GBS” section, we get the time complexity for classically simulating a limited connected GBS process as stated in the following Theorem 2.

#### Theorem 2

A limited connected GBS process with a bandwidth *w* can be simulated in $O(M nw2^w)$ time, where *M* represents the number of optical modes and *n* denotes the maximum total photon number of the output samples.

#### Proof

The classical simulation process is similar to that in Ref.^14^, as summarized in “Classical simulation of GBS” section. In this process, we sequentially sample $n_k$ for $k=1,\dots ,M$ according to Eq. (24). As we shown in Theorem 1, the computation of Eq. (24) takes at most $O\left( n w 2^w \right)$ steps, assuming that *B* has a bandwidth *w*, where $n = \sum _{i=1}^{M} n_i$. This is scaled up by at most the total number of modes *M*. Thus the time complexity for simulating such a GBS process is $O\left( M n w 2^w \right)$. $\square$

### Validity of the algorithm

By sequentially computing the states (usually non-Gaussian) of the remaining $M-k$ modes with the measurement outcome of modes 1 to *k* ($k=1,\dots ,M$) in photon number basis, we can demonstrate the validity of the algorithm introduced in “Loop Hafnian algorithm for banded matrices” section. Recalling matrix ${\tilde{R}}$ defined in Eq. (15), we denote the bandwidth of matrix ${\tilde{R}}$ by *w*. According to the definition of ${\tilde{R}}$ and ${\tilde{l}}$, we know that

31 $$\begin{aligned} \mathrm{lhaf}(R) = \mathrm{lhaf}(A), \end{aligned}$$

where

32 $$\begin{aligned} R_{i,j} = {\left\{ \begin{array}{ll} ({\tilde{R}})_{i, j} &{}\quad {\text{if }} i \ne j \\ {\tilde{l}}_i &{}\quad {\text{if }} i = j \end{array}\right. }. \end{aligned}$$

Assuming that the measurement results are $n_i = 1$ for every $i = 1, \dots , M$, we obtain

33 $$\begin{aligned} p(11 \dots 1) = {\mathscr {P}}_0 \mathrm{lhaf}(R). \end{aligned}$$

Although we make the assumption that *R* corresponds to a Gaussian state, the subsequent proof remains valid in the more general case as discussed in Appendix C.

For convenience, define ${\tilde{\gamma }}_{d_i} = (\beta _i^*,\alpha _i)^\mathrm{T}$, ${\tilde{\gamma }}_{h_i} = (\beta _{i+1}^*,\alpha _{i+1},\dots ,\beta _{M}^*,\alpha _{M})^\mathrm{T}$, ${\tilde{R}}_{dd}^i$ as a $2\times 2$ matrix corresponding to mode *i*, ${\tilde{R}}_{hh}^i$ as a $2(M-i) \times 2(M-i)$ matrix corresponding to modes $i+1$ to *M*, ${\tilde{R}}_{dh}^i$ as a $2 \times 2(M-i)$ matrix represents the correlation between mode *i* and modes $i+1$ to *M*.

According to Eq. (17), after measuring the mode 1 in photon number basis, the remaining non-Gaussian quantum state is:

34 $$\begin{aligned} \begin{aligned} {\hat{\rho }}^{(n_1)}&= \frac{{\mathscr {P}}_0}{\pi ^{2(M-1)}} \int \mathrm{d}^2 \vec {\alpha }_{(1)} \int \mathrm{d}^2 \vec {\beta }_{(1)} |\vec {\beta }_{(1)}\rangle \langle \vec {\alpha }_{(1)} | \\ {}&\quad \times \frac{\partial ^2}{\partial \alpha _1 \partial \beta _1^*} {\exp } \left( \frac{1}{2} {\tilde{\gamma }}_{d_1}^\mathrm{T} {\tilde{R}}_{dd}^1 {\tilde{\gamma }}_{d_1} +{\tilde{\gamma }}_{d_1}^\mathrm{T} {\tilde{R}}_{dh}^1 {\tilde{\gamma }}_{h_1} + {\tilde{\gamma }}_{d_1}^\mathrm{T}{\tilde{l}}_{d_1}\right) \Bigg |_{\gamma _{d_1}=0}\\&\quad \times {\exp } \left( -\frac{1}{2}|{\tilde{\gamma }}_{h_1}|^2 + \frac{1}{2} {\tilde{\gamma }}_{h_1}^\mathrm{T} {\tilde{R}}_{hh}^1 {\tilde{\gamma }}_{h_1} + {\tilde{\gamma }}_{h_1}^\mathrm{T}{\tilde{l}}_{h_1}\right) . \end{aligned} \end{aligned}$$

We then have

35 $$\begin{aligned} \begin{aligned}{}&\frac{\partial ^2}{\partial \alpha _1 \partial \beta _1^*} {\exp } \left( \frac{1}{2} {\tilde{\gamma }}_{d_1}^\mathrm{T} {\tilde{R}}_{dd}^1 {\tilde{\gamma }}_{d_1} + {\tilde{\gamma }}_{d_1}^\mathrm{T} {\tilde{R}}_{dh}^1 {\tilde{\gamma }}_{h_1} + {\tilde{\gamma }}_{d_1}^\mathrm{T}{\tilde{l}}_{d_1}\right) \Bigg |_{\gamma _{d_1}=0} \\ {}&\quad = \left( {\tilde{R}}_{dd}^1 \right) _{1,2} + \left( {\tilde{l}}_{d_1}\right) _1\left( {\tilde{l}}_{d_1}\right) _2 + \sum _{j,k=1}^{M} \left( {\tilde{R}}_{dh}^1\right) _{1,j} \left( {\tilde{R}}_{dh}^1\right) _{2,k} \left( {\tilde{\gamma }}_{h_1}\right) _j \left( {\tilde{\gamma }}_{h_1}\right) _l \\&\quad = {\tilde{R}}_{12} + {\tilde{l}}_1 {\tilde{l}}_2 + + \sum _{j,k=1}^{M} {\tilde{R}}_{1,j+2} {\tilde{R}}_{2,k+2} {\tilde{\gamma }}_{j+2} {\tilde{\gamma }}_{k+2}\\&\quad = C^2_{\emptyset } + \sum _{Z^2} C^2_{Z^2} \prod _{x \in Z^2} {\tilde{\gamma }}_x + \sum _{x \in \{3,\dots ,M\} } D^2_x {\tilde{\gamma }}_x^2, \end{aligned} \end{aligned}$$

where $D^2_x$ is the coefficient for ${\tilde{\gamma }}_x^2$.

Next, measuring mode 2. The remaining non-Gaussian quantum state is:

36 $$\begin{aligned} \begin{aligned} {\hat{\rho }}^{(n_1n_2)}&= \frac{{\mathscr {P}}_0}{\pi ^{2(M-2)}} \int \mathrm{d}^2 \vec {\alpha }_{(2)} \int \mathrm{d}^2 \vec {\beta }_{(2)} |\vec {\beta }_{(2)}\rangle \langle \vec {\alpha }_{(2)} | \\&\quad \times {\exp } \left( -\frac{1}{2}|{\tilde{\gamma }}_{h_2}|^2 + \frac{1}{2} {\tilde{\gamma }}_{h_2}^\mathrm{T} {\tilde{R}}_{hh}^2 {\tilde{\gamma }}_{h_2} + {\tilde{\gamma }}_{h_2}^\mathrm{T}{\tilde{l}}_{h_2}\right) \\&\quad \times \frac{\partial ^2}{\partial \alpha _2 \partial \beta _2^*} \left[ {\exp } \left( \frac{1}{2} {\tilde{\gamma }}_{d_2}^{\mathrm{T}} {\tilde{R}}_{dd}^2 {\tilde{\gamma }}_{d_2} +{\tilde{\gamma }}_{d_2}^{\mathrm{T}} {\tilde{R}}_{dh}^2 {\tilde{\gamma }}_{h_2} + {\tilde{\gamma }}_{d_2}^\mathrm{T}{\tilde{l}}_{d_2}\right) \right. \\&\quad \left. \left. \times \left( C^2_{\emptyset } + \sum _{Z^2} C^2_{Z^2} \prod _{x \in Z^2} {\tilde{\gamma }}_x + \sum _{x \in \{3,\dots ,M\} } D^2_x {\tilde{\gamma }}_x^2\right) \right] \right| _{\gamma _{d_2}=0}. \end{aligned} \end{aligned}$$

We have

37 $$\begin{aligned} \begin{aligned}{}&\frac{\partial ^2}{\partial \alpha _2 \partial \beta _2^*} \left[ {\exp } \left( \frac{1}{2} {\tilde{\gamma }}_{d_2}^\mathrm{T} {\tilde{R}}_{hh}^2 {\tilde{\gamma }}_{d_2} +{\tilde{\gamma }}_{d_2}^\mathrm{T} {\tilde{R}}_{dh}^2 {\tilde{\gamma }}_{h_2} + {\tilde{\gamma }}_{d_2}^\mathrm{T}{\tilde{l}}_{d_2}\right) \right. \\&\qquad \left. \left. \times \left( C^2_{\emptyset } + \sum _{Z^2} C^2_{Z^2} \prod _{x \in Z^2} {\tilde{\gamma }}_x + \sum _{x \in \{3,\dots ,M\} } D^2_x {\tilde{\gamma }}_x^2\right) \right] \right| _{\gamma _{d_2}=0}\\&\quad = C^4_{\emptyset } +\sum _{Z^4} C^4_{Z^4} \prod _{x \in Z^4} {\tilde{\gamma }}_x +\sum _{x_1,x_2,x_3 \in \{5,\dots ,M\}} D^4_{x_1,x_2,x_3} {\tilde{\gamma }}_{x_1}^2 {\tilde{\gamma }}_{x_2} {\tilde{\gamma }}_{x_3} ,\\ \end{aligned} \end{aligned}$$

where $D^4_{x_1,x_2,x_3}$ is the coefficient for ${\tilde{\gamma }}_{x_1}^2 {\tilde{\gamma }}_{x_2} {\tilde{\gamma }}_{x_3}$.

Repeating this procedure to measure mode 1 to mode *M*, we eventually find that

38 $$\begin{aligned} {\hat{\rho }}^{({\bar{n}} = 11\dots 1)} = {\mathscr {P}}_0 C^{2M}_\emptyset = p({\bar{n}} = 11\dots 1) = {\mathscr {P}}_0 \mathrm{lhaf}(R) . \end{aligned}$$

Thus we prove

39 $$\begin{aligned} C^{2M}_\emptyset = {\text{lhaf}}(R). \end{aligned}$$

This demonstrates the validity of the algorithm given in “Loop Hafnian algorithm for banded matrices” section.

Intuitively, as shown in the previous derivation, after measuring the first *t* modes of the output Gaussian state, the state of the remaining modes is:

40 $$\begin{aligned} \begin{aligned} {\hat{\rho }}^{(n_1\dots n_t)}&= \frac{{\mathscr {P}}_0}{\pi ^{2(M-t)}} \int \mathrm{d}^2 \vec {\alpha }_{(t)} \int \mathrm{d}^2 \vec {\beta }_{(t)} |\vec {\beta }_{(t)}\rangle \langle \vec {\alpha }_{(t)} | \\&\quad \times {\exp } \left( -\frac{1}{2}|{\tilde{\gamma }}_{h_t}|^2 + \frac{1}{2} {\tilde{\gamma }}_{h_t}^\mathrm{T} {\tilde{R}}_{hh}^t {\tilde{\gamma }}_{h_t} + {\tilde{\gamma }}_{h_t}^\mathrm{T}{\tilde{l}}_{h_t}\right) \\&\quad \times \mathrm{Poly}^t \left( R,{\tilde{\gamma }}_{h_t} \right) ,\\ \end{aligned} \end{aligned}$$

where $\mathrm{Poly}^t \left( R,{\tilde{\gamma }}_{h_t} \right)$ represents the sum of polynomial terms formed by complex variables in ${\tilde{\gamma }}_{h_t}$. Specifically, we have

41 $$\begin{aligned} \begin{aligned} \mathrm{Poly}^t (R,{\tilde{\gamma }}_{h_t})&= C^{2t}_\emptyset + \sum _{Z^{2t}} C^{2t}_{Z^{2t}} \prod _{x \in Z^{2t}} {\tilde{\gamma }}_x \\&\quad +\sum _{x_1,\dots ,x_{2t-1} \in \{2t+1,\dots ,M\}} D^{2t}_{x_1,\dots ,x_{2t-1}} \\&\quad \times {\tilde{\gamma }}_{x_1}^2 {\tilde{\gamma }}_{x_2} \dots {\tilde{\gamma }}_{x_{2t-1}}. \end{aligned} \end{aligned}$$

First we consider the case where $t<M$. It is easy to find that terms $\{\prod _{x \in Z^{2t}} {\tilde{\gamma }}_x\}$ of different $Z^{2t}$ influence the constant term $C^{2M}_\emptyset$ in the subsequent computing. On the other hand, high-order terms containing $\gamma _x^k$ with $k\ge 2$ do not affect the final result. As those term vanishes after performing the partial derivation. Consequently, the iteration of $C^t_{Z^t}$ appearing in step 3 of our algorithm is required from $t=1$ to $t=2M$ to eventually determine the value of $\mathrm{lhaf}(R) = C^{2M}_\emptyset$. When all modes of the output state are measured, we have $t=M$ for Eq. (41), and hence $\mathrm{Poly}^{M} (R,{\tilde{\gamma }}_{h_M})$ will not contain any complex variables, ensuring that the constant term $C^{2M}_\emptyset$ equals to $\mathrm{lhaf}(R)$.

## Summary

To verify quantum advantage, it is vital to evaluate the concrete cost of classically simulating the corresponding quantum process. Here we present an algorithm to speed up the classical simulation of GBS with limited connectivity. The speed-up is arising from the faster calculation for the loop Hafnian of banded (or sparse) matrices. This algorithm runs in $O\left( nw2^w\right)$ for $n\times n$ symmetric matrices with bandwidth *w*. This result is better than the prior state-of-the-art result of $O\left( nw^2 2^w\right)$^18^.

This classical algorithm is helpful on clarifying how limited connectivity reduces the computational resources required for classically simulating GBS processes, thereby tightening the boundary for achieving quantum advantage in GBS problem.

### Supplementary Information

Supplementary Information.

## Data Availability

All data generated or analysed during this study are included in this published article.
