# Supplementary material for: Speeding up the classical simulation of Gaussian boson sampling with limited connectivity
Source: Sci Rep. 2024 Apr 1;14:7680. doi: 10.1038/s41598-024-58136-1 (PMC10984997; doi:10.1038/s41598-024-58136-1)
Supplement: Supplementary file 1 — Supplementary Information. [file 41598_2024_58136_MOESM1_ESM.pdf]

A Example

Here we give an example of computing the loop Hafnian of a 4×4 symmetric matrix with bandwidth $w = 1$ by the algorithm given in Sec. 3.1. We consider the following case:

$$\begin{aligned}\mathrm{lhaf}\,(A) = &\mathrm{lhaf}\left(\begin{bmatrix} A_{1,1} & A_{1,2} & 0 & 0 \\ A_{2,1} & A_{2,2} & A_{2,3} & 0 \\ 0 & A_{3,2} & A_{3,3} & A_{3,4} \\ 0 & 0 & A_{4,3} & A_{4,4} \end{bmatrix}\right) \\ =&A_{1,1}A_{2,2}A_{3,3}A_{4,4} + A_{1,1}A_{2,2}A_{3,4} \\ &+ A_{1,1}A_{2,3}A_{3,4} + A_{1,2}A_{3,3}A_{4,4} \\ &+ A_{1,2}A_{3,4}.\end{aligned} \tag{1}$$

The algorithm given in Sec. 3.1 works as follows:
Step 1.
Let $C_{\emptyset}^{0} = 1$.
Step 2.
Let $C_{\{2\}}^{1} = A_{1,2}$ and $C_{\emptyset}^{1} = A_{1,1}$.
Step 3.
Let $C_{\{3\}}^{2} = A_{2,3}C_{\emptyset}^{1} = A_{1,1}A_{2,3}$ and $C_{\emptyset}^{2} = A_{2,2}C_{\emptyset}^{1} + C_{\{2\}}^{1} = A_{1,1}A_{2,2} + A_{1,2}$.
Step 3.
Let $C_{\{4\}}^{3} = A_{3,4}C_{\emptyset}^{2} = A_{1,1}A_{2,2}A_{3,4} + A_{1,2}A_{3,4}$ and $C_{\emptyset}^{3} = A_{3,3}C_{\emptyset}^{2} + C_{\{3\}}^{2} = A_{1,1}A_{2,2}A_{3,3} + A_{1,2}A_{3,3} + A_{1,1}A_{2,3}$.
Step 4.
Let $C_{\emptyset}^{4} = A_{4,4}C_{\emptyset}^{3} + C_{\{4\}}^{3} = A_{1,1}A_{2,2}A_{3,3}A_{4,4} + A_{1,2}A_{3,3}A_{4,4} + A_{1,1}A_{2,3}A_{4,4} + A_{1,1}A_{2,2}A_{3,4} + A_{1,2}A_{3,4}$.
We can see that $C_{\emptyset}^{4} = \mathrm{lhaf}\,(A)$.

B Loop Hafnian of sparse matrices

It is easy to find that, with slight modifications, the algorithm given in Sec. 3.1 can be used to compute the loop Hafnian of symmetric sparse matrices. Denote the largest number of zero-valued entries in each rows of a sparse matrix B as w. The modified algorithm is outlined as follows.
Algorithm. To calculate the loop Hafnian of an $n \times n$ symmetric sparse matrix B with at most w non-zero entries in each rows:

1. Let $C_{\emptyset}^{0} = 1$.

For $t = 1, \ldots, n$:

2. Let w_t be the number of non-zero entries in row t. Let $\{t_1, \ldots, t_{w_t}\}$ be the columns that $B_{t,t_i} \neq 0$ for $i = 1, \ldots, w_t$, and $P(\{t_1, \ldots, t_{w_t}\})$ be the set of all subsets of $\{t_1, \ldots, t_{w_t}\}$.
3. For every $Z^t \in P(\{t_1, \ldots, t_{w_t}\})$ satisfying $Z^t \neq \emptyset$ and $|Z^t| \leq \min(t, w_t)$, if $t_{w_t} \in Z^t$, then

$$C_{Z^t}^{t} = \sum_{x \in Z^t} B_{t,x} C_{Z^t \setminus \{x\}}^{t-1} + C_{Z^t \cup \{t\}}^{t-1} + B_{t,t} C_{Z^t}^{t-1}, \tag{2}$$

   and if $t_{w_t} \in Z^t$, then

$$C_{Z^t}^{t} = B_{t,t_{w_t}} C_{Z^t \setminus \{t_{w_t}\}}^{t-1}. \tag{3}$$

   During the above iterations, if $C_{\{\ldots\}}^{t-1}$ is not given in the previous steps, it is treated as 0.
4. Let

$$C_{\emptyset}^{t} = B_{t,t} C_{\emptyset}^{t-1} + C_{\{t\}}^{t-1}. \tag{4}$$

The loop Hafnian of matrix B is obtained in the final step $t = N$ by

$$\mathrm{lhaf}\,(B) = C_{\emptyset}^{n}. \tag{5}$$

The time cost for this algorithm is $O\left(nw2^w\right)$.

C Validity of the loop Hafnian algorithm for arbitrary matrix R

For an arbitrary even matrix R, as shown in Ref.[1,2], we have

$$\prod_{i=1}^{M}\left(\frac{\partial^2}{\partial\alpha_i\partial\beta_i^*}\right)\exp\left[\frac{1}{2}\tilde{\gamma}^{\mathrm{T}}\tilde{R}\tilde{\gamma}+\tilde{\gamma}^{\mathrm{T}}\tilde{l}\right]\Bigg|_{\tilde{\gamma}=0}=\mathrm{lhaf}(R). \tag{6}$$

If we calculate the partial derivative for $i=1,\ldots,t$, we have

$$\begin{aligned}\mathrm{lhaf}(R)=&\prod_{i=t+1}^{M}\left(\frac{\partial^2}{\partial\alpha_i\partial\beta_i^*}\right)\mathrm{Poly}^t(R,\tilde{\gamma}_{h_t})\\&\times\exp\left[\frac{1}{2}\tilde{\gamma}_{h_t}^{\mathrm{T}}\tilde{R}_{hh}^{t}\tilde{\gamma}_{h_t}+\tilde{\gamma}_{h_t}^{\mathrm{T}}\tilde{l}_{h_t}\right]\Bigg|_{\tilde{\gamma}_{h_t}=0}.\end{aligned} \tag{7}$$

For an arbitrary odd matrix R with rank $2M+1$, we have

$$\prod_{i=1}^{M}\left(\frac{\partial^2}{\partial\alpha_i\partial\beta_i^*}\right)\frac{\partial}{\partial\beta_{M+1}^*}\exp\left[\frac{1}{2}\tilde{x}^{\mathrm{T}}\tilde{R}\tilde{x}+\tilde{x}^{\mathrm{T}}\tilde{z}\right]\Bigg|_{\tilde{\gamma}=0}=\mathrm{lhaf}(R), \tag{8}$$

where $\tilde{x}=(\tilde{\gamma}^{\mathrm{T}},\beta_{M+1}^*)^{\mathrm{T}}$, $\tilde{z}=(\tilde{l}^{\mathrm{T}},\tilde{l}_{2M+1})^{\mathrm{T}}$. If we calculate the partial derivative for $i=1,\ldots,t$, we have

$$\begin{aligned}\mathrm{lhaf}(R)=&\prod_{i=t+1}^{M}\left(\frac{\partial^2}{\partial\alpha_i\partial\beta_i^*}\right)\frac{\partial}{\partial\beta_{M+1}^*}\mathrm{Poly}^t(R,\tilde{\gamma}_{h_t})\\&\times\exp\left[\frac{1}{2}\tilde{x}_{h_t}^{\mathrm{T}}\tilde{R}_{hh}^{t}\tilde{x}_{h_t}+\tilde{x}_{h_t}^{\mathrm{T}}\tilde{z}_{h_t}\right]\Bigg|_{\tilde{x}_{h_t}=0},\end{aligned} \tag{9}$$

where $\tilde{x}_{h_t}=(\beta_{t+1}^*,\alpha_{t+1},\ldots,\beta_{M+1}^*)^{\mathrm{T}}$ and $\tilde{z}_{h_t}=(\tilde{l}_{2t+1},\tilde{l}_{2t+2},\ldots,\tilde{l}_{2M+1})^{\mathrm{T}}$.

As shown in Eq. (7) and (9), the analysis in Sec. 3.2 is valid for any symmetric matrix R.

References

1. Hamilton, C. S. *et al.* Gaussian Boson Sampling. *Physical Review Letters* **119**, 170501 (2017). URL `https://link.aps.org/doi/10.1103/PhysRevLett.119.170501`.
2. Kruse, R. *et al.* Detailed study of Gaussian boson sampling. *Physical Review A* **100**, 032326 (2019). URL `https://link.aps.org/doi/10.1103/PhysRevA.100.032326`.
